# True pattern-reversal LED stimulator and its comparison to LCD and CRT displays: visual evoked potential study

**DOI:** 10.1038/s41598-024-54776-5

**Published:** 2024-02-20

**Authors:** P. Voda, J. Kremláček, D. Kordek, M. Chutná, A. Bezrouk

**Affiliations:** 1 Department of Medical Biophysics, Medical Faculty in Hradec Kralove, Charles University, Simkova 870, 500 03 Hradec Kralove, Czech Republic; 2 Department of Pathological Physiology, Medical Faculty in Hradec Kralove, Charles University, Simkova 870, 500 03 Hradec Kralove, Czech Republic

**Keywords:** Electrical and electronic engineering, Electronics, photonics and device physics, Sensory processing, Visual system

## Abstract

A rapid checkerboard pattern change is used to elicit pattern-reversal visual evoked potentials (PR VEPs). CRT or LCD monitors do not allow immediate reversal of the entire pattern. The study aimed to construct a new stimulator whose characteristics approximate an instantaneous reversal and verify whether the improvement is reflected in PR VEPs. A new stimulator using a matrix of 12 × 48 independent white square LEDs was designed and compared with LCDs and CRTs. The effect on the PR VEP peak times and amplitudes of N70, P100, and P140 waves was evaluated in ten subjects. The LED stimulator showed significantly better performance in the rate of change of illuminance, change of pattern, luminance settling and stability. The PR VEP amplitudes of N75, P100, and N140 did not show significant differences. The sum of interpeak amplitudes was significantly larger for the LCD than for the other monitors. The peak times of the waves evoked by the LED were shorter than those evoked by the LCD and CRT for the N75 wave and a check size of 30´. LED stimulators are a better alternative to CRTs for PR VEPs than current LCDs. LEDs also seem to be better than CRTs, but further research is necessary to obtain significant results.

## Introduction

Visual evoked potentials (VEPs) are electrical potentials produced by averaging the epochs of the electroencephalogram recorded in response to an optical stimulus. VEPs are used to quantify the functional integrity of a visual analyzer, including the retina, optic nerves, nerve pathways, and visual cortex. VEPs are of significant diagnostic benefit, particularly for diseases such as multiple sclerosis and retrobulbar optic neuritis. VEP abnormalities accompany optic nerve gliomas in neurofibromatosis, compression of the optic pathways due to hydrocephalus or pituitary tumor, etc. The optical stimulus for VEP examination is, for example, inversion of contrast elements of the imaged structure. Usually, this structure is a black and white chessboard whose squares are interchanged over time. This process is referred to as "pattern-reversal visual stimulation", and the VEPs are referred to as “pattern-reversal VEPs” (PR VEPs)^1–2^. To make the results of different laboratories comparable, the International Society for Clinical Electrophysiology of Vision (ISCEV) has created a standard defining the properties of imaging systems. This ISCEV standard defines the properties of the imaging pattern, display times, contrast, luminance, etc.^3,4^.

The first stimulator for PR VEP stimulation was a classical mechano-optical system (Maxwellian view system), consisting of a light source (xenon lamp) and a tilting mirror system. Today, a computer monitor connected to an electronic generator of a variable checkerboard structure (alternatively a computer or a part of a separate VEP registration device) is used. The gold standard for the computer monitor was the CRT screen, which consists of a glass vacuum tube in which an electron beam hits the screen and causes luminophores to light up ^1^. However, as CRT monitors have gradually disappeared from the computer market, neurophysiology laboratories are trying to replace the CRT screens with the currently available flat panel displays equipped with liquid crystal layers (LCDs, TFTs) ^5–7^ or organic light-emitting diodes (OLEDs)^8–10^ or even with projectors^11^.

Both the technical parameters and the principle of image formation of current imaging systems can affect the parameters of the VEPs^6,10,12–15^. The main imperfection common to all aforementioned imaging systems is the time it takes for the structure to reverse across the entire display area. The mirror of the mechano-optical system has a finite reversal time (on the order of ms), the beam in a CRT rasterizes and sequentially illuminates the individual luminophore elements (with a frequency of 50–100 Hz), and the time for the crystals in an LCD to rotate is also finite (ms). Thus, in all the above mentioned types of devices, the image is gradually formed, and the reversal of the whole structure takes time, the duration of which is comparable to the transmission characteristics of the human visual system, thus affecting the obtained results^6,15^.

This led to an attempt to replace a commercial screen with an array of light emitting diodes LEDs^15^. However, the design of the published simulator was limited to a very small display array (10 × 10 LEDs) with a single type of projected pattern. The LEDs used were round, separated by baffles and covered by a diffuse screen, which degraded the homogeneity of the luminance of a single checkerboard square, decreased the contrast between the illuminated and nonilluminated areas of the checkerboard pattern, and caused different sizes of the light and dark elements. The described design weaknesses affected the resulting evoked potentials, particularly their lower amplitude^15^.

Based on the abovementioned studies^6,10,12–15^, we set the hypothesis that a suitable LED simulator design providing faster and synchronous image generation, i.e., a short reversal time, could have a beneficial effect on the peak time, amplitude, or width of the PR VEP peaks. Note, in the context of this text, the term “LED” refers to a discrete optical element, representing a single component that emits light independently, not flat panel displays like MicroLED.

The aim of our study was to develop a new type of stimulator based on white LEDs that would have no inertia, regularly square and closely spaced image elements, no baffle between the elements and no diffuse sheet preventing interaction among the image elements. The stimulator should produce the image all at once in the whole area, not sequentially (by rasterization).

Consequently, we opted to compare the parameters of our essentially brand-new VEP stimulator with the CRT monitor (HP p1230) utilized for VEP examinations in our electrophysiology lab and a standard LCD monitor (Acer V176Lb). Our selection of the CRT monitor was based on its frequent use in electrophysiological vision testing^7,16,17^, while the LCD monitor was chosen because LCDs are increasingly replacing no longer available CRTs. We did not assess other currently available display technologies, as they are not frequently employed for electrophysiological examinations. Furthermore, these alternatives have limitations, such as time-dependent contrast deterioration and image burning, and have already undergone comprehensive testing^9^. The objective was to investigate whether stimulator's significantly improved temporal characteristic is reflected in the VEPs of experimental subjects.

## Methods

### Examination and ethical approval

The experiment was carried out in a dark, electromagnetically shielded examination cabin of the electrophysiological laboratory at the Department of Pathological Physiology, Faculty of Medicine in Hradec Králové, Charles University, Czech Republic, in accordance with the standards,^3^ on a sample of 10 people (3 men, 7 women, aged 34–56 years). Each investigated person signed informed and GDPR consent forms prior to the examination. All procedures carried out in our study were in accordance with the Institutional Commission's ethical standards and with the 1964 Declaration of Helsinki and its later amendments or comparable ethical standards. The study was approved by the Ethics Committee, University Hospital Hradec Králové, Czech Republic (No. 202002S14P).

### VEP recording

Before the experiment, each person was inquired about vision-related or neural diseases. Only those who did not report any visual or neurological problems participated in the study. Prior to the VEP examination, we determined the refractive error of both eyes in each examined person (NIDEK ARK-1A AUTOREFRACTOMETER, NIDEK CO., LTD., Gamagori, Japan). The dominant eye, through which the subject subsequently observed the stimulators, was determined on the basis of the lower equivalent refractive error. If it was impossible to determine the dominant eye in this way, we used the Dolman method (hole-in-card test)^18^. To eliminate the effects of fatigue, the order of individual stimulators and the viewing distance were chosen pseudorandomly.

We registered the EEG using Ag–AgCl sintered electrodes with a conductive paste (Ten20) with an impedance below 10 kΩ at the following positions: Oz, Fz, and four electrodes placed in a cross, top, bottom, left and right, 5 cm from the Oz electrode. The reference electrode was placed on the left ear (A1), and the ground electrode was placed on the left wrist. The signal was recorded in the frequency range from 0.3 to 100 Hz with a sampling rate of 3000 Hz.

The recording was made by a TruScan electroencephalograph (Alien technik /Deymed, Hronov, Czech Republic). For each type and setting of the stimulator, 2 measurements were made so that each resulting VEP curve was obtained by averaging 2 × 100 stimuli.

### VEP stimuli

Stimulation by reversing a chessboard with a square edge of 0.5 cm was used to induce PR VEPs. The frequency of stimulation was 2 reversals per second. The viewing distances of 1160 mm and 580 mm were chosen by moving the examined person so that one element of the structure was observed at an angle of 15′ or 30′, respectively.

Three stimulators (LCD, CRT and LED) were used for the experiment and were placed close to each other in the electrophysiology laboratory cabin. A common mask was applied to the stimulators. Three coverable equally sized rectangular holes of 240 × 60 mm were cut in the mask to define the same stimulation field on each stimulator. For the individual monitors, the delay from the trigger to the actual rise of luminance in the upper left corner of the display area defined by the mask was corrected for. While the LED stimulator had its own control system (see below), the LCD and CRT monitors were sequentially connected to a ViSaGe MKII stimulus generator (Cambridge Research Systems Ltd, UK).

Luminance measurements were performed in all cases using a Tektronix J16 photometer with a J6503 measuring probe (Tektronix Inc., Beaverton, OR, USA) attached to the display surface and verified with photometer Minolta LS 160 (Konica Minolta Inc., Tokyo, Japan).The luminance of the white element of the reversible structure was set to meet mean luminance (calculated along ISCEV calibration standard^19^) 50 Cd/m^2^ on all displays^3^. The central white element's median luminance (Cd/m^2^) was 100 for CRT, 99 for LCD, and 101 for LED. The black element's median luminance (Cd/m^2^) was 1.5 for CRT, 0.1 for LCD, and lower than 0.1 (below the photometer resolution) for LED. The corresponding Michelson contrast was 97% for CRT, 99.8% for LCD, and above 99.8% for LED.

### Testing of technical parameters of stimulators

The technical parameters of the stimulators were tested under the same conditions (in the dark cabin of the laboratory at the Department of Pathological Physiology) as those during the examination of the VEP test subjects. The measurements were performed by a probe with a BPW21R photodiode (Vishay Intertechnology, Inc. Malvern, PA, USA) with an output loaded with a 1.5 kΩ resistor, as in the studies by Zhang^5^, Naggy^6^, and Cooper^8^. The probe was connected to the first input channel of a Rigol 2012 oscilloscope (RIGOL Technologies Co., Ltd., Suzhou, China) and placed in the upper left corner of the stimulator without the mask so that it scanned one square element of the reversible structure.

We compared the stimulators in terms of (i) the rise time of the luminance to 80% of the maximum value (the white part of reversible structure set to 100 Cd/m^2^)^3^, (ii) the luminance fluctuations greater than 10% after reaching the maximum value, and (iii) the luminance decline time to drop luminance to 10% of its maximum value. The evaluation was performed on the oscilloscope recordings by reading the voltage value representing the luminance.

A synchronization pulse (trigger) for VEP recording indicating the reversal of the structure was connected to the second channel of the oscilloscope to measure the synchronicity of the trigger with the light output. The time parameters were related to the ascending edge of the trigger.

To eliminate PR VEP peak time differences caused by the cartoon mask position and stimulator construction, we measured the delay between the trigger and the structure reversal in the upper left corner of the mask.

### CRT stimulator

An HP p1230 monitor (Hewlett-Packard, Palo Alto, CA, USA)—a professional 22" CRT monitor with a Trinitron TCO03 screen, an 800 × 600/60 Hz resolution, and Diamondtron Natural Flat (NF) technology—was used.

### LCD stimulator

An Acer V176Lb monitor (Acer, New Taipei, Taiwan)—a 17" monitor for office work with a resolution of 800 × 600/60 Hz, up to 16.77 million colors, and a 5 ms response time—was used.

### LED stimulator

We created a new LED stimulator composed of discrete LED elements. Regarding temporal properties, these LED elements adhere to the requirement of rapid emission changes. Each element can be independently controlled, facilitating true pattern-reversal. Beyond achieving a swift simultaneous reversal of the entire stimulus area, our design focuses on creating a checkerboard pattern with high contrast among and uniform luminance within all elements. The elements should be of a square radiation area, positioned closely to each other, and engineered to avoid luminous interference. Our considerations also include ease of assembly within our electrophysiology laboratory constraints. To meet these criteria, we opted for commercially produced square LEDs with a diffusing emitting surface of 5 × 5 mm.

Considering the technical parameters of the electronic components used, easier repair of possibly defective parts, and the possibility of creating display devices of any size while maintaining the reversal time of the image structure, we chose a modular concept of the stimulator display.

**Figure 1:**
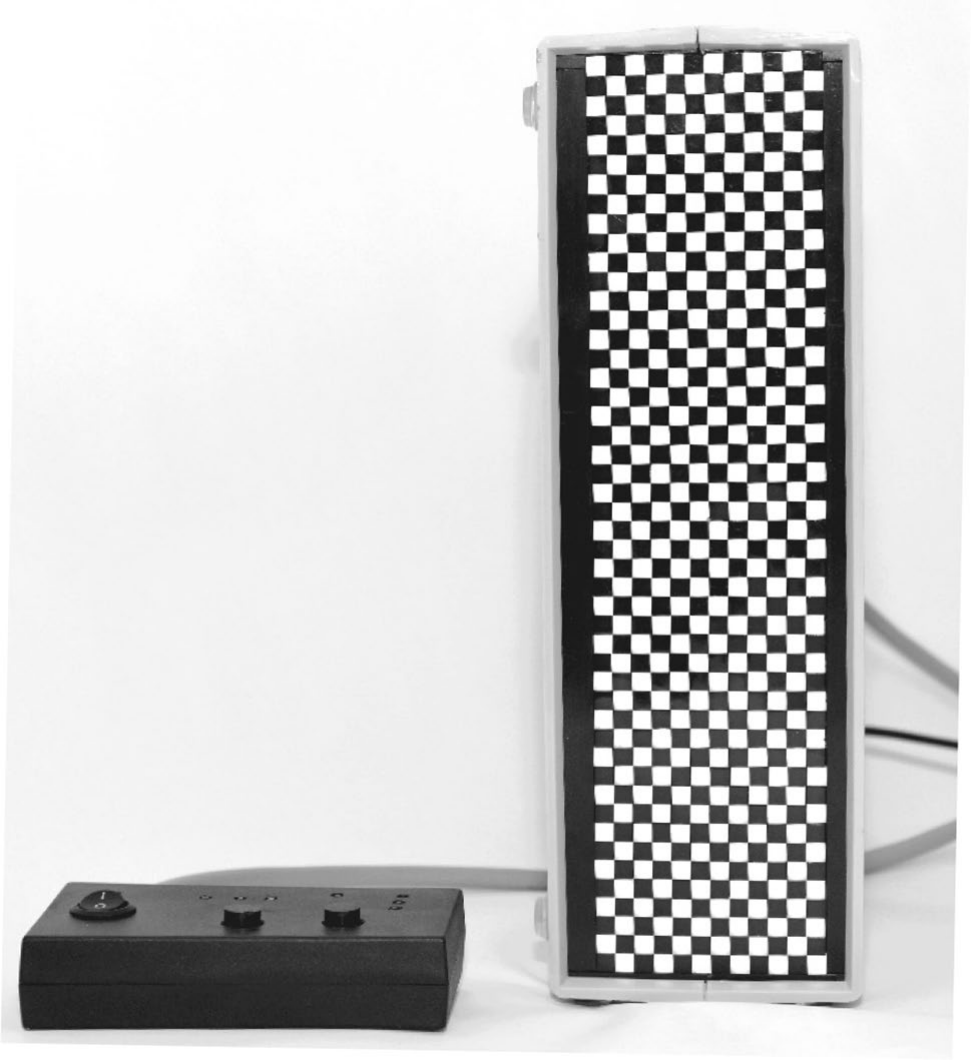
Photo of a running LED stimulator with a control board.

The LED stimulator used in this study (Fig. 1) consisted of four modules with common controls, forming a vertical strip with an image size of 60 × 240 mm (12 × 48 LEDs).

**Figure 2:**
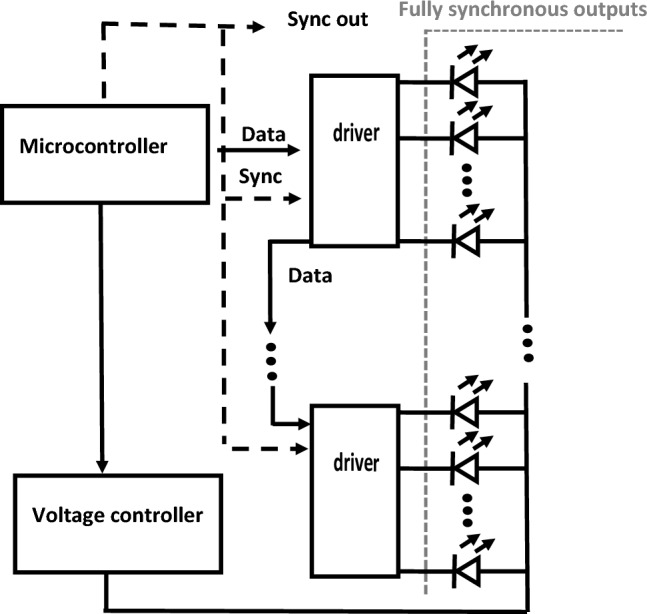
Block schematic of the LED stimulator.

A single module itself consisted of 144 matte white-light LED elements with a flat matte square face with a 5 mm edge (THT LED OSW5YK7NE2B, Optosupply, Hong Kong) and a controller (74HC595T, Diodes Incorporated, Plano, TA, USA). The diodes along with the controller form a compact block with dimensions of 60 × 60x50 mm (12 × 12 LEDs). The LEDs were placed close together without gaps. Each LED was covered on its five sides with black paint to achieve the highest possible opacity and contrast between the elements. The cathode of each LED was connected (1:1) to a separate pin of the controller array. Each LED was therefore separately controlled (Fig. 2). Other components of the LED stimulator included a linear regulated power supply to provide stabilization and brightness control of the LEDs and a system of three switchable red fixation points with a diameter of 0.5 mm. The resulting display was controlled by an Arduino Nano v 3.0 microcomputer (Arduino, Monza, Italy), in which the individual display structures were stored in a programmable manner, alternating with an adjustable period. Any structure (black and white images) could be created from basic square elements with a 5 mm edge. These structures were fed into the controller in series. It took 2 ms to load one structure. The reversal of the whole structure at once was then achieved by a single command to all parallelly controlled LEDs.

### Statistics and data analyses

The evaluation of the measured PR VEPs was performed using a TruScan EEG Explorer (Deymed diagnostic s.r.o, Hronov, Czech Republic). The following parameters were measured: the absolute amplitude of the N75, P100 and N140 waves in µV, the peak time of the N75, P100 and N140 waves in ms, and the width of the P100 peak (w-P100), defined as the time span of the peak when the amplitude decreases to half of the peak value. Because the on-screen mask delimits only a part of the screen, the peak time data were corrected for the measured delay between the actual luminance rise in the upper left corner of the delimited part of the stimulus image and the trigger.

Data were processed using MS Excel 2016 (Microsoft Corp., Redmond WA, USA). Statistical analysis was performed in RStudio (2023.3.0.386), R version 4.2.3^20^ with the rstatix library^21^. The parameters describing the peak time, peak width, and interpeak amplitude were compared via a two-factor (monitor and element size) analysis of variance with repetition. The normality of the data was checked using a Q‒Q plot. Mauchly's test was used to test sphericity, and Greenhouse‒Geisser correction was applied in case of violation. A paired t test with Bonferroni correction for multiple comparisons was used for post hoc tests. The significance level, alpha, was 5% for all statistical comparisons.

In the whole set of assessed parameters, we cannot reject the data coming from a normal distribution. Since in three out of 42 tests, the Shapiro‒Wilk test was below 5% (but not less than p corrected for multiple comparisons), we used the median and the first and third quartiles for data description.

## Results

### Technical parameters of stimulators

**Figure 3:**
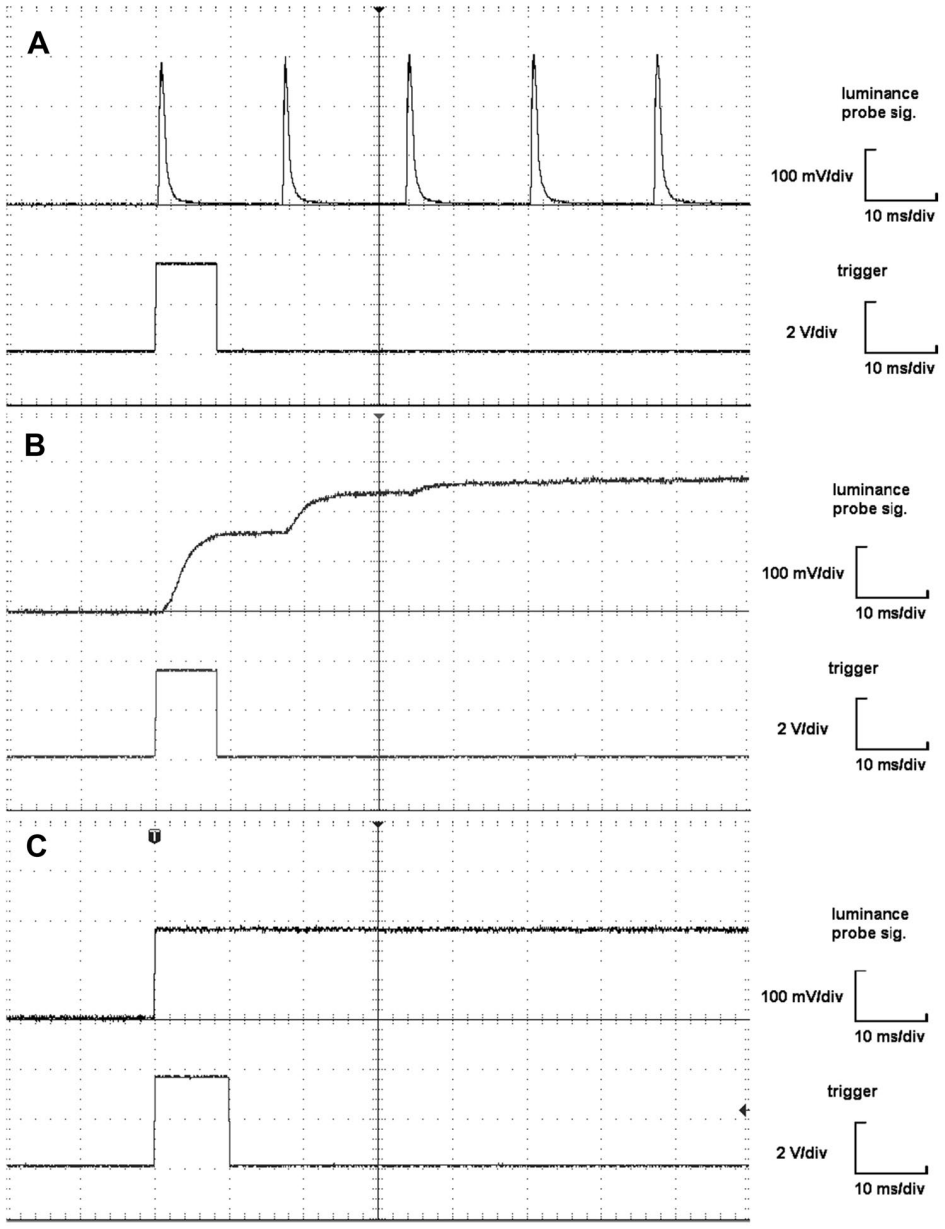
(**A–C**) Luminance onset of the measured CRT and LCD monitors and LED stimulator. Every panel shows an oscilloscope recording of the upper-left corner of the (**A**) CRT, (**B**) LCD, and (**C**) LED displays. In each print screen, the top channel shows the voltage representing the luminance reversal of the image element. The reversal signal sent by the stimulator is indicated by the leading edge of the trigger in the bottom channel. The time scale is 10 ms per division. The LED display shows superior luminance onset and stability compared to the flashing CRT and slow onset of the LCD.

**Figure 4:**
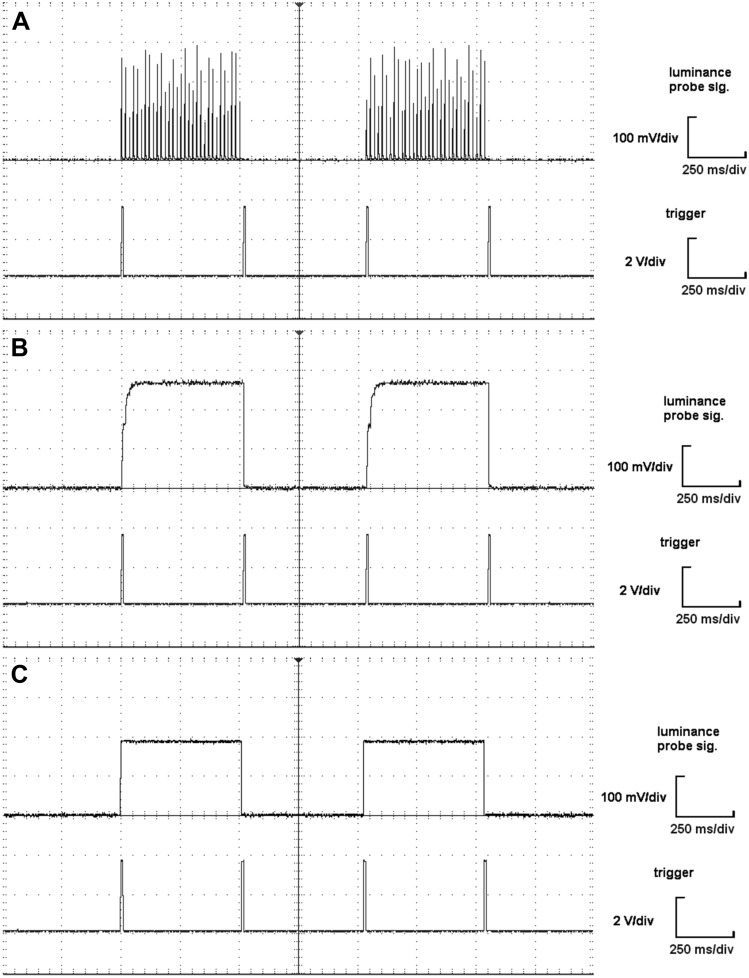
(**A–C**) Comparison of the luminance variation of each display. Every panel shows four luminance reversals recorded in the upper-left corner of the (**A**) CRT, (**B**) LCD, and (**C**) LED displays. In each oscilloscope print screen, the top channel shows the voltage representing the luminance reversal of the image element. The reversal indication sent by the stimulator is indicated by the leading edge of the trigger in the bottom channel. The time scale is 250 ms per division. The LED display shows superior luminance onset, offset, and stability compared to the flashing CRT and the slow onset of the LCD.

For all the tested monitors, the rise times, luminance fluctuations, and luminance decline times were determined from the oscilloscope records, as depicted in Figs. 3 and 4.

#### CRT

The luminance of the white image element is not stable and regularly fluctuates (luminance fluctuates more than 10%) (Fig. 3A), with a luminance fluctuation period of approximately 17 ms (Fig. 4A), corresponding to the set image refresh rate of 60 Hz. The luminance rise time of a single spot to 80% luminance is approximately 1.3 ms from the leading edge of the trigger, and the luminance decline time is approximately 2 ms (Fig. 4A).

#### LCD

The measurements of the LCD monitor show (Fig. 4B) that the luminance rise of the white element of the structure is gradual. The detailed time evolution (Fig. 3B) shows two phases. The lightening of the element starts approximately 1 ms after the leading edge of the trigger and reaches approximately 60% luminance in 8 ms. A subsequent increase in luminance to 80% takes another 15 ms. Thus, it takes a total of approximately 24 ms to light up the reversible structure element (Fig. 4B). The dimming of the image element is significantly faster, and the luminance decline time is 1 ms. No luminance fluctuation is noticeable after reaching the full luminance throughout the lighting period.

#### LED

The LED stimulator has substantially better technical parameters than the compared LCD and CRT monitors. The brightening characteristics of the LED element (see Fig. 3C) reveal an initial delay of 3 µs, with the rise time to reach 80% of the luminance maximum within another 3 µs (after the initial delay). The luminance decline time is equally rapid. It's worth noting that the real-time characteristics might be even shorter, considering the measuring photodiode BPW21R's limitations (rise time 3.1 µs, fall time 3 µs^22^). Given that the LED timing is three orders of magnitude faster than both LCD and CRT technologies, we accepted the inherent uncertainty introduced by the photodiode limitations.

The LEDs are driven by the 74HCT595 controller, which boasts control pulse processing times in the tens of nanoseconds (propagation delay 17 ns, max. 35 ns, enable time 17 ns, max. 30 ns^23^). Consequently, the total delay in this circuit is less than 100 ns. This suggests that the reversal time is primarily dictated by the LED elements' speed, a factor three orders of magnitude superior to the measured CRT and LCD stimulators.

Compared to the CRT monitor, the luminance of the white element is stable, and no luminance fluctuation is noticeable throughout the lighting period (Fig. 3A, C).

The measurements were repeated 50 times, and we did not observe any variations in the trigger-to-monitor response delay for any of the stimulators.

### VEP

#### Peak times

**Figure 5:**
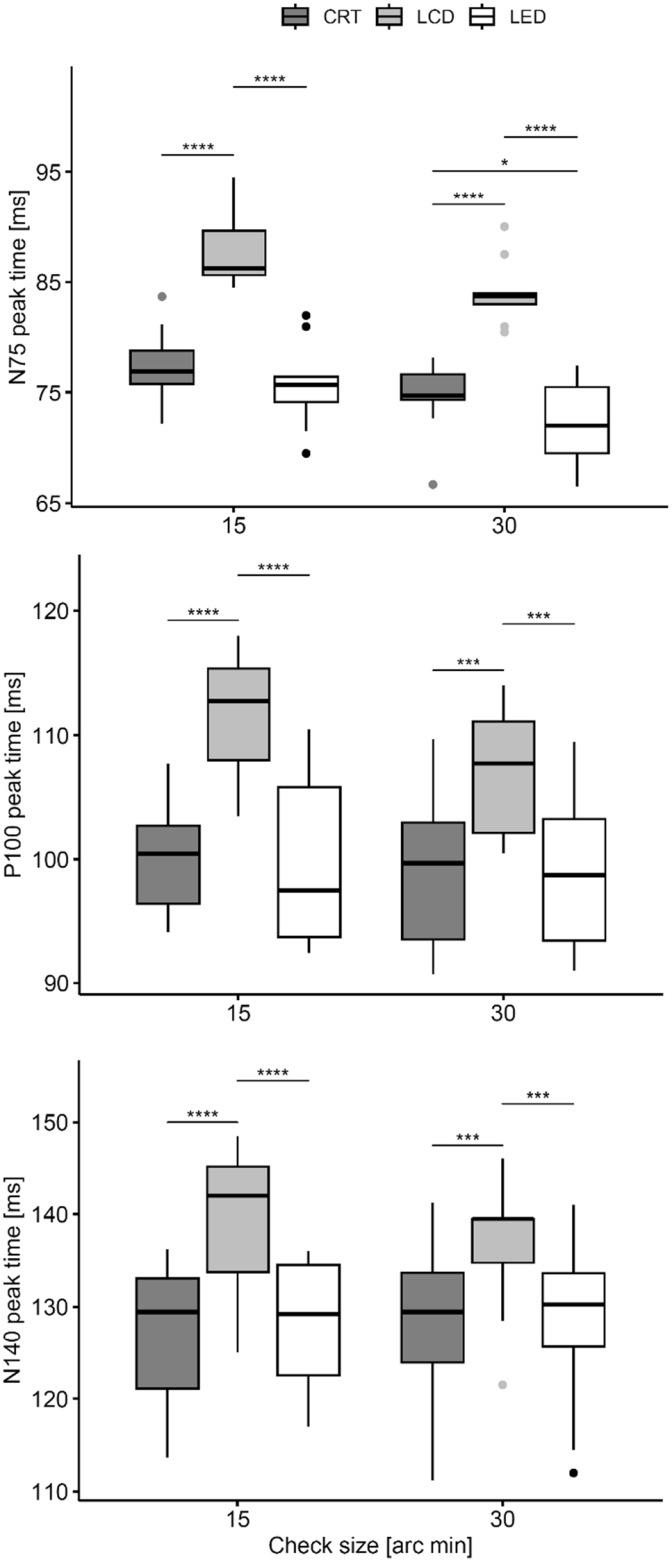
Comparison of the PR VEP peak times (upper graph—N75 wave, middle graph—P100 wave, and bottom graph N140 wave) measured for individual displays—CRT, LCD, and LED. For stimulation patterns with PR 30′, they are grouped on the right, and for patterns with PR 15′, they are grouped on the left part of every graph. Measures of significant differences are indicated by asterisks (*).

The results of the peak time measurements are shown in Fig. 5. The measured values for the N75 and P100 peaks for all stimulators corresponded to normal physiological values ^1^. The display type is a significant factor for all three peak times, F(2, 18) > 71.6, p < 0.001, generalized eta squared > 0.29. Post hoc analyses using paired t tests with Bonferroni adjustment reveal that the peak times are significantly (p < 0.001) shorter for the LED and CRT stimulators than for the LCD monitor (by approximately 15 ms). Compared to the CRT monitor, the difference for the LED is significant (p = 0.044) only for the N75 wave and the check size of 30´ (by 3 ms).

**Figure 6:**
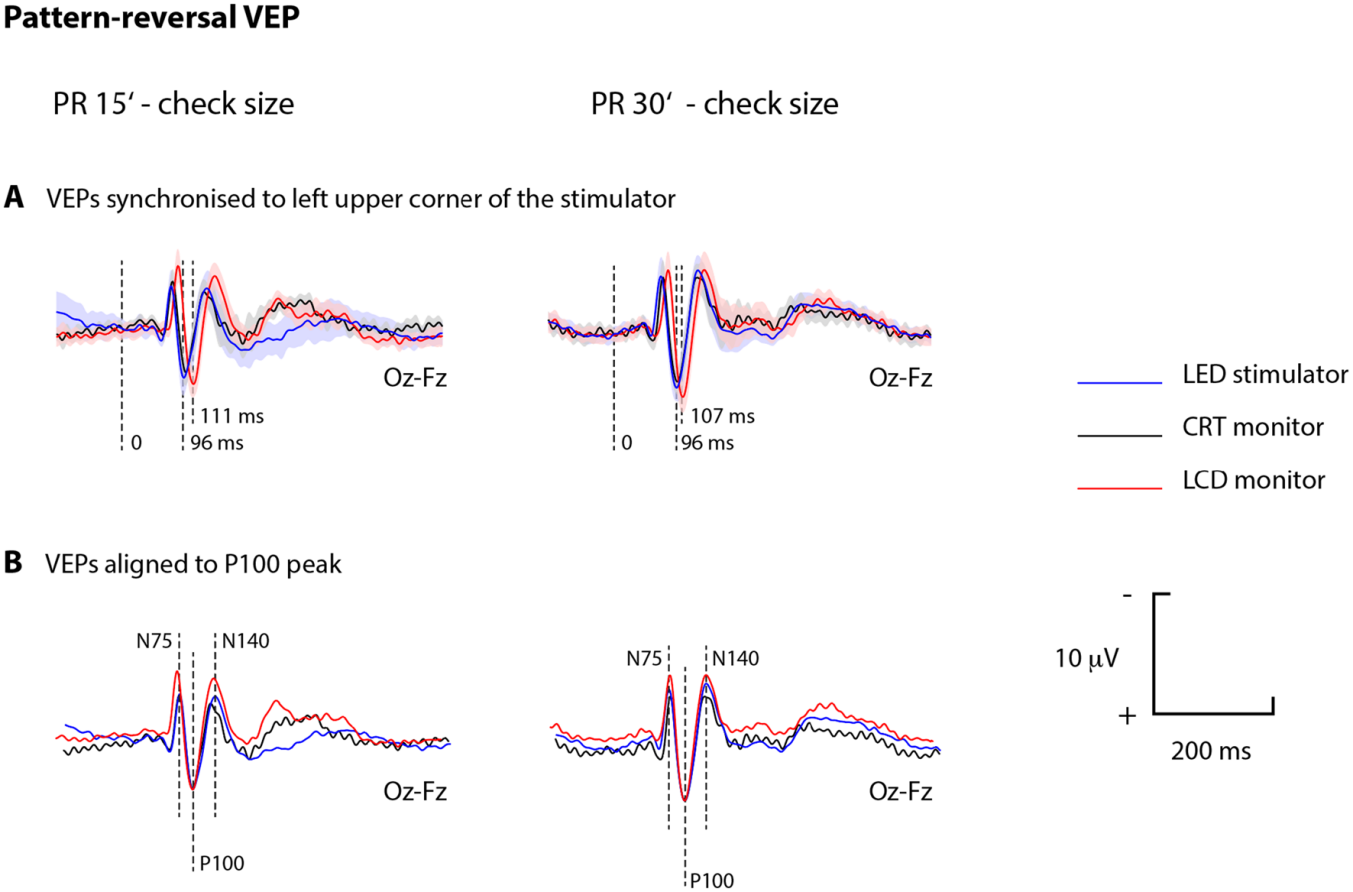
Group mean PR VEP. Responses to reversal stimulation averaged over two repetitions; all subjects are shown by the blue, black, and red curves for the LED, CRT, and LCD stimulators, respectively. The semitransparent patches under curves depicts 95% CI for the VEPs. PR 30′ VEPs are grouped on the right and PR15′ VEPs on the left side of the figure. At the top of the figure (**A**), the PR VEPs are aligned to the onset of reversal of each stimulus detected in the upper left corner of the stimulated area. This instant is marked as "0". In the lower part of the figure (**B**), the same PR VEPs are aligned to the P100 peak. Note the low amplitude noise for the black curves. This noise was created by the electromagnetic field of the CRT monitor at its frame rate (60 Hz).

Additionally, a graphical comparison of PR VEPs averaged over two repetitions and all subjects shows that for both angular sizes of the reversal structure, the responses evoked by the LCD stimulator have longer peak times (Fig. 6).

#### Amplitudes

**Figure 7:**
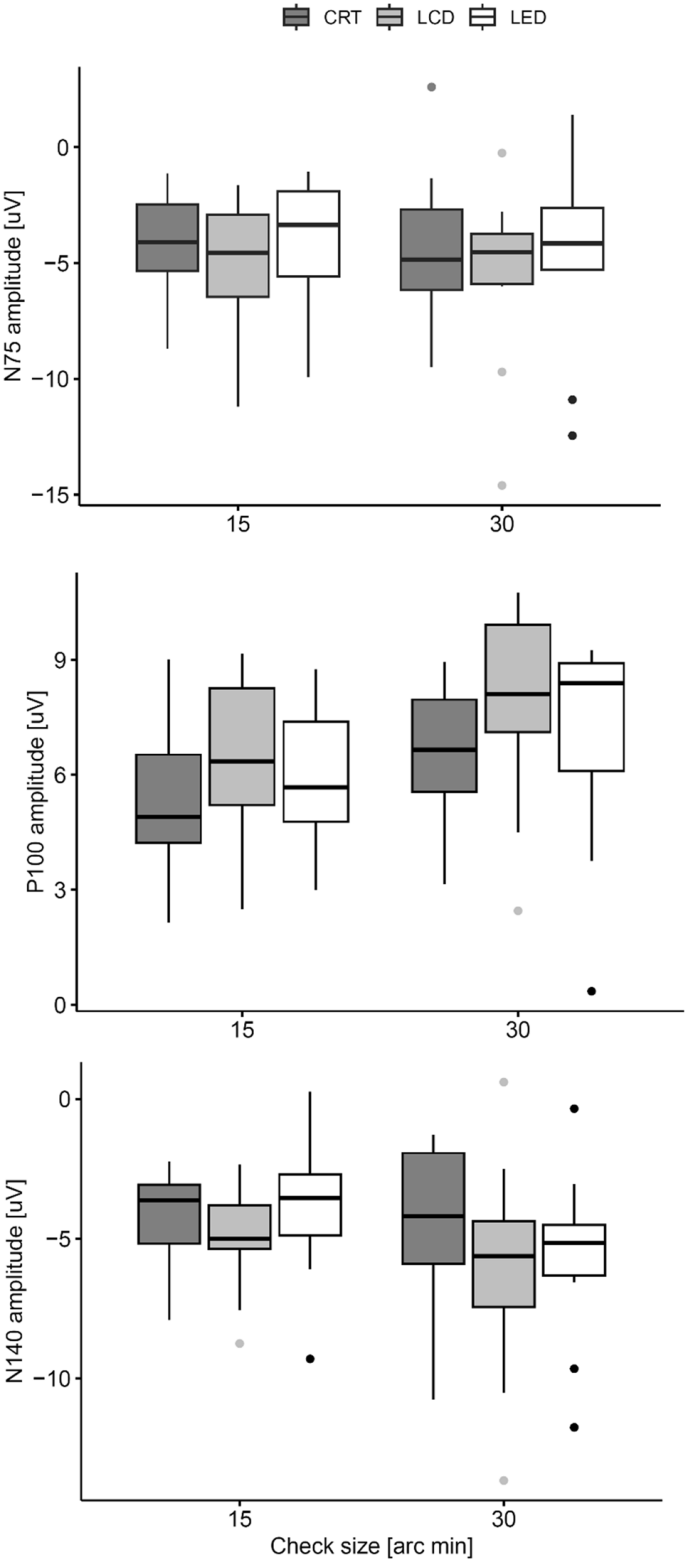
Comparison of the PR VEP amplitudes measured for individual displays—CRT, LCD, and LED. For stimulation patterns with PR 30′, they are grouped on the right, and for patterns with PR 15′, they are grouped on the left of the figure.

The results of the peak amplitude measurements for the N75, P100 and N140 peaks are shown in Fig. 5. For each amplitude, there are no significant differences (F(2, 18) < 3.5, p > 0.051, generalized eta squared < 0.05) among the stimulators. Despite the failure to show a significant difference in the absolute peak amplitudes (Fig. 7), it is evident from the graphical comparison (PR VEPs aligned to P100, Fig. 6) that for both the PR 15' and PR 30' structures, the interpeak amplitudes are more prominent for the LCD stimulator than for the other two stimulators. This led us to perform a post hoc ANOVA of the sum of interpeak amplitudes (P100-N75 + P100-N145), which showed that the interpeak amplitudes are different (F(2, 18) = 7.8, p = 0.004, generalized eta squared < 0.105) and that the LCD monitor evokes a larger response (p < 0.012). There is no significant difference between the responses to the LCD and CRT stimulation (p = 0.43).

#### P100 wave peak width

**Figure 8:**
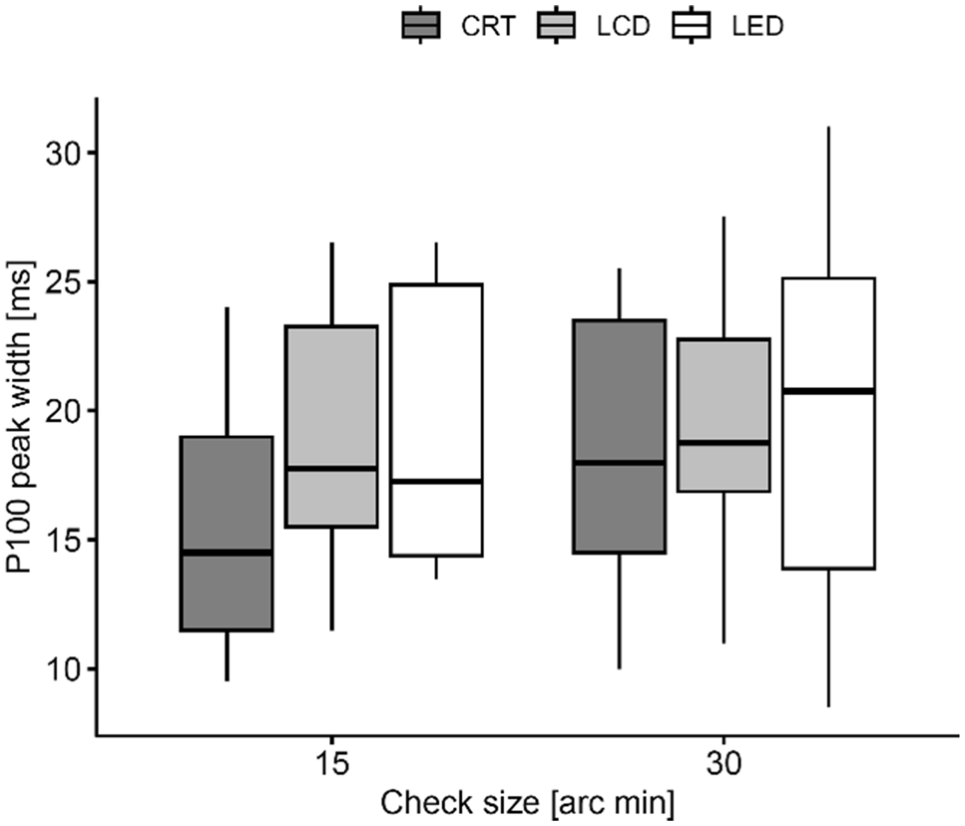
Comparison of the P100 "peak width" for different displays—CRT, LCD, and LED. They are grouped on the right and on the left of the figure for the stimulation patterns with PR 30′ and PR 15′, respectively.

The results of the w-P100 measurements are shown in Fig. 8. No significant differences are observed among the stimulators, which is obvious from the graphical comparison of the PR VEPs aligned to P100 in Fig. 6.

## Discussion

The PR VEP examination is a useful visual function diagnostic method. However, the peak times (N75, P100 and N140) depend on the technical parameters of the stimulator (display unit) used. A number of studies describe these dependencies for different types of display units (LCD/CRT: ^5–7,17^; LCD: ^14,24^; OLED: ^8,10^; curved OLED: ^9^; DLP: ^11^) and different aspects of their use^6,14,16^.

The results of the abovementioned studies suggest that such devices that can provide the imaged structure at once over the entire area, with high spatial and temporal homogeneity of imaging, can improve the quality of stimulation and thus the PR VEPs obtained.

The CRT and LCD do not fulfill these expectations, as documented by the temporal profiles of the luminance measured (Figs. 3A, B and 4A, B). The temporal profile of a single screen spot illustrates the principles of image formation. On a CRT screen, the electron beam travels across the screen, causing its points to light up and fade out when the beam moves to other pixels. To keep the apparent lighting of a particular pixel, the beam repeatedly activates the pixel, creating a flicker. This manifests as sharp, short (approximately 2 ms) peaks in the oscilloscopic recording of the luminance (Fig. 3A). The LCD monitors light up its points by rotating liquid crystals, which changes their opacity to the luminous background. The process of rotating the crystals is slower, so the luminance onset is gradual (23 ms), but the point does not flicker (see Fig. 3B). The temporal profiles obtained for the CRT monitor (Figs. 3A, 4A) and the LCD monitor (Figs. 3B, 4B) are consistent with the results of previous studies^5,7,8,25^.

In addition to the lighting up of a single point, the displays also differed in the way of changing the entire pattern. The CRT and LCD monitors draw the checkerboard from the top to the bottom, line by line, so the last line of the display is drawn with a delay (for our CRT monitor, this delay is approximately 13 ms; the delay is determined by the vertical and horizontal frequency of the monitor and, in our experiment, also by size of the mask). Such a rasterizing approach is also adopted for OLED displays, which are recommended as an alternative to LCD and CRT monitors for vision science^8^. The rasterization limits the possibility of producing an instant pattern change, and the time for reversal is orders of magnitude longer for the OLED^10^ as well as for our CRT and LCD monitors than for the LED stimulator reported here (3 µs), which renders the whole pattern simultaneously. We achieved this by independently controlling the LED elements. Such a device more closely approaches an ideal pattern-reversal stimulation than CRT, LCD, or OLED monitors. There have been previous attempts to use LEDs for pattern-reversal stimulation^15,26,27^. However, the constructed stimulators had some limitations.

An LED stimulator composed of 8 × 8 rounded red LEDs^27^ was successfully used for PR VEP elicitation^28^, but the rounded LEDs were assumed to be a reason for the small VEP amplitude. Epstein et al.^26^ used 108 red, high-brightness rectangular LEDs arranged in 6 columns and 18 rows, with reversal times under 100 µs. Such a stimulator was successfully used for a clinical evaluation and exhibited a significantly shorter P100 peak time and a lower amplitude than the checkerboard pattern generated using a television with a 60 Hz refresh rate^29^. Although the red LED PR VEPs were shown to have a higher and partially independent sensitivity in diagnosing multiple sclerosis^30^, the black‒white checkerboard was accepted as the standard stimulus (ISCEV 2016). Link et al.^15^ used 100 white LEDs for stimulation with colors corresponding to the ISCEV standard. The authors confirmed that the PR VEPs elicited by their LED stimulator correspond to those elicited by CRT and Maxwellian stimulators. However, the structure did not completely match the checkerboard because there was a grid between the elements to prevent light diffusion between the elements. This grid created a 9′ gap between elements and did not change luminance with the reversal. The size of the stimulation element was 69′.

Compared to the aforementioned LED stimulators, our display produced instant reversal with a black and white checkerboard of high contrast (100%). The checkerboard had dimensions close to those of the clinically used patterns (we used element sizes of 15' and 30') and a larger visual field (12 × 48 LEDs corresponding to 6° × 24° for PR 30' and 3° × 12° for PR 15'). The junctions between LEDs were tight and did not exceed 1'. Modifying the LED matrix by painting the walls of each LED element with an opaque black color ensured minimal crosstalk (Fig. 1). This is a great improvement over the problems caused by the shielding baffles and the scattering surface of the stimulator studied by Link^15^. Separating the round LEDs with paper baffles with a paper overlay causes less-dark black areas and crosstalk between the chambers (an unsharp interface between light and dark spots) and thus low contrast of the imaged structure^15^.

For instant reversal, a fast rise time of the luminance at a single point and a quick drawing of the pattern in the whole screen are necessary. Our LED stimulator showed significantly better transient characteristics than the CRT and LCD we tested. The LED stimulator had three orders of magnitude faster luminance rise and fall (3 µs—this value was at the boundary of the light probe used). The time of pattern drawing (ca. 16.6 ms at an ~ 60 Hz vertical frequency) dominated the CRT reversal time, as the rise time was 0.6 ms. (The rise time depended on the position and size of the light detector, which can partially explain the rise time we measured. A probe with a diameter of 1 cm was placed in the upper left corner of the display, and for a vertical monitor size of 30 cm, a vertical frequency of 60 Hz can register a delay of 0.3 ms.). On the other hand, the LCD reversal time dominated the gradual rise time of the white element luminance (23 ms to 80% luminance), which was longer than the time for drawing the pattern (16.6 ms).

At the end of the technical discussion, we should mention that the VEP response depends on pattern generation and pattern-reversal synchronization with the EEG recording. Our experiment used a standalone stimulus generator for the CRT and LCD monitors with high synchronization accuracy between the video and trigger. Generally, PC-based stimulus generators may have a higher or lower synchronization precision^31^.

We compared two types of monitors, an LCD and a CRT, with our custom-designed LED stimulator to determine whether the faster transient parameters of the LED stimulator would affect the characteristics of evoked potentials. In our comparison, we used the same stimulation areas for all displays with identical element sizes and overall luminance. Furthermore, we corrected the recordings for the delay between the trigger and the beginning of the reversal in the display.

We confirmed the hypothesis that the short reversal time of the LED stimulator (3 µs), compared to the CRT (16 ms—caused mostly by the sequential rendering) and LCD (23 ms—caused mostly by the rise time), displays significantly reduced peak times. When comparing the LED and LCD (Fig. 4), significant differences were observed for all waves (N75, P100 and N140) and check sizes (15' and 30'), but this was mainly due to the characteristics of the LCD monitor itself^6,15^. This finding is consistent with the results of the comparison between a CRT and an LCD investigated by Husain et al. (Husain et al. 2009; Baumgarten et al. 2022) showing that the LCD causes an increase in the peak time of both the N75 and P100 waveforms due to a longer luminance rise time. A significant difference between the CRT and LCD was observed only for the N75 wave and PR 30´.

The influence of the tested technical parameters on the P100 wave width was not confirmed (Fig. 8), nor did the post hoc analysis of the time interval from the N75 to N140 peak show any significant change (p > 0.217).

We did not observe differences in peak amplitude values among the stimulators for the N75, P100, and N140 peaks (Fig. 5). However, the group average curves (Fig. 6) showed higher responses for the LCD stimulation. Post hoc, we evaluated the cumulative amplitude of all three amplitudes. In ANOVA, the monitor was a significant factor for (p = 0.004) the N75-P100-N140 complex amplitude. The complex amplitude was significantly the largest for the LCD in paired comparison to the LED (p = 0.012) or CRT (p < 0.001) stimulation. The reason for such superiority of the LCD might be caused by the checkerboard luminance homogeneity and its sharp edges. However, this hypothesis cannot be verified by our data.

Our study has some strengths and some limitations. We developed an LED stimulator that, among the stimulators compared, comes closest in its characteristics to the ideal of instantaneous reversal of the structure. The LED stimulator proved to be a possible replacement for the "gold standard" CRT. It has better technical parameters compared to both the CRT and LCD and appears to be an equal or better method for PR VEP stimulation. Our LED stimulator design complies with the ISCEV standard. The modular design of the stimulator makes it possible to expand the display field to almost any shape and size. The presented LED stimulator construction did not produce electromagnetic interference with the VEPs, as the CRT monitor did (see Fig. 6).

Due to the extremely fast image rendering and high luminance stability, the presented stimulator also allows for the exploration of fast transient phenomena, such as the flickering observed in a CRT or DLP, or the simulation of the gradual luminance rise of LCD monitors.

However, the LED stimulator and the test methods used in this study have some limitations. *Some parameters of our LED stimulator do not comply with ISCEV standards, as shown the *Table 1*.* Due to the need to reduce the average luminance of the structure to 50 Cd/m^2^ to achieve the ISCEV standard, the LEDs of the stimulator were operated at the very low end of their luminance range, which caused the luminance of the white element to not be perfectly homogeneous across its entire area. There is room for design improvements in this respect. Increasing the luminance (to a level where the whole area is already homogeneously illuminated) and placing a thin foil in front of the display area (attenuating the luminance according to the ISCEV standard without undesirable crosstalk among LED elements) is being considered.

**Table 1:** Parameters of LED display and how they match the ISCEV standard.

	ISCEV standard	LED stimulator	Comply
Stimulus type	Pattern reversal	Pattern reversal	OK
Field size (minimum)	15°	6° × 24° resp 3° × 12°	X
Presentation	Monocular	Monocular	OK
Stimulus: check widths	0.25° resp 1°	0.25° resp. 0.5°	OK resp X
Mean luminance (Cd/m^2^)	50 (40–60)	50	OK
Michaelson contrast (%)	≥ 80	100	OK
Presentation rate (rev/s)	2	2	OK
Viewing distance (cm)	50–150	58 resp. 116	OK
Checks shape	Square	Square	OK
Number of light/dark checks	Equal	Equal	OK
Aspect ratio width/ height	Max 4:3	1:4	X
Narrowest dimension of field	Min 15°	6° resp. 3°	X
Fixation point	At corners	At corners	OK
Transient luminance change	No	No	OK

Another limitation arises when trying to display different sizes of squares. The fixed LED size (5 × 5 mm) allows a homogeneous display of elements of the checkerboard of the LED size or multiples thereof. For squares that are multiples of the LED size, the gap between the LEDs is visible in the element. Therefore, we varied the viewing distance in our experiment to avoid distortion. Another limitation that may have influenced the results is the small horizontal size of the imaging area of our LED stimulator prototype. We intentionally used a vertically extended stimulation area since we expected that the vertical rendering of the CRT and LCD might cause sequential activation and a broader P100 peak, which we did not find.

## Conclusions

We designed and built, in principle, an entirely new VEP stimulator based on white LEDs approaching the parameters of the ISCEV standard^3^. The LED stimulator shows better technical parameters compared to the currently commonly used LCD and CRT monitors in terms of the rate of rise and fall of the luminance of the reversible structure element (by three orders of magnitude—80% luminance rise in 3 µs) and luminance stability.

The LED stimulator we designed, unlike the LCD and CRT, allows for instantaneous reversal of the structure in the entire display area at once. This makes it possible to investigate the effect of fast transients on the VEP.

We have shown that the peak times of the N75, P100 and N140 waves evoked by the LED stimulator are shorter than those evoked by the LCD stimulator (significantly for all waves and check sizes of 15´ and 30´) and by the CRT stimulator (significantly only for the N75 wave and check size of 30´). Comparison of the absolute N75, P100, and N140 wave amplitudes showed no significant differences among these stimulators; however, the cumulative N75-P100-N140 amplitude showed slightly higher values for LCD stimulation.

The LED stimulator we developed is a more suitable alternative to CRTs than the LCD stimulators currently used for the examination of visual evoked potentials. The LED stimulator also appears to be superior to the CRT stimulator. Further development to improve the stimulator luminance homogeneity is possible.

## Data Availability

The datasets generated during and/or analyzed during the current study are available from the corresponding author on reasonable request.

## Abbreviations

VEP: Visual evoked potentials
LED: Light-emitting diode
CRT: Cathode ray tube
LCD: Liquid crystal display
PR 15′: 15′ Visual angle of one element of a checkerboard pattern
PR 30′: 30′ Visual angle of one element of a checkerboard pattern
